# On the Cluster Formation of α-Synuclein Fibrils

**DOI:** 10.3389/fmolb.2021.768004

**Published:** 2021-10-19

**Authors:** Marija Dubackic, Ilaria Idini, Veronica Lattanzi, Yun Liu, Anne Martel, Ann Terry, Michael Haertlein, Juliette M. Devos, Andrew Jackson, Emma Sparr, Sara Linse, Ulf Olsson

**Affiliations:** ^1^ Division of Physical Chemistry, Department of Chemistry, Lund University, Lund, Sweden; ^2^ Division of Biochemistry and Structural Biology, Department of Chemistry, Lund University, Lund, Sweden; ^3^ Center for Neutron Research, National Institute of Standards and Technology, Gaithersburg, MD, United States; ^4^ Chemical and Biomolecular Engineering Department, University of Delaware, Newark, DE, United States; ^5^ Institut Laue-Langevin, Grenoble, France; ^6^ ISIS Neutron and Muon Source, Harwell Oxford, Didcot, United Kingdom; ^7^ Max IV Laboratory, Lund University, Lund, Sweden; ^8^ Life Sciences Group, Institut Laue-Langevin, Grenoble, France; ^9^ European Spallation Source, Lund, Sweden

**Keywords:** alpha-synuclein, amyloid fibril, fractal cluster, Lewy bodies (LB), small-angle neutron scattering (SANS), rigid-rod cluster modeling

## Abstract

The dense accumulation of α-Synuclein fibrils in neurons is considered to be strongly associated with Parkinson’s disease. These intracellular inclusions, called Lewy bodies, also contain significant amounts of lipids. To better understand such accumulations, it should be important to study α-Synuclein fibril formation under conditions where the fibrils lump together, mimicking what is observed in Lewy bodies. In the present study, we have therefore investigated the overall structural arrangements of α-synuclein fibrils, formed under mildly acidic conditions, pH = 5.5, in pure buffer or in the presence of various model membrane systems, by means of small-angle neutron scattering (SANS). At this pH, α-synuclein fibrils are colloidally unstable and aggregate further into dense clusters. SANS intensities show a power law dependence on the scattering vector, *q*, indicating that the clusters can be described as mass fractal aggregates. The experimentally observed fractal dimension was *d* = 2.6 ± 0.3. We further show that this fractal dimension can be reproduced using a simple model of rigid-rod clusters. The effect of dominatingly attractive fibril-fibril interactions is discussed within the context of fibril clustering in Lewy body formation.

## 1 Introduction

Amyloids are protein-rich fibrillar aggregates that possess a characteristic β-sheet structure (Serpell, Berriman et al., 2000; Jahn, Makin et al., 2010). Their presence constitutes the hallmark for several related neurodegenerative diseases, including Parkinson’s, Alzheimer’s disease, and type II diabetes (Spillantini and Goedert 2000; Ghiso and Frangione 2002). The association of amyloid fibrils with various diseases has led to extensive research in the field of amyloid fibrils (Chiti and Dobson 2006; Eisenberg and Jucker 2012; Iadanza, Jackson et al., 2018; Ke, Zhou et al., 2020). Despite extensive studies, the link between the amyloid fibril formation and pathology is still unclear in several of these diseases, and therapies are just starting to emerge (Tanzi 2021).

The morphology and composition of the amyloid deposits vary among different diseases and may also vary for the same disease (Tycko 2015). Therefore, understanding the structural and chemical properties of the amyloid aggregates is highly relevant as the structural features of the amyloid deposits may carry information on the process and conditions that lead to their formation and may serve as a basis for therapeutic discoveries. This motivates detailed and systematic investigations of amyloid deposits formed under different conditions.

One protein that has received much interest in amyloid-related research is α-Synuclein, αS, associated with a group of overlapping neurodegenerative disorders called α-synucleinopathies (Spillantini and Goedert 2000; Visanji, Lang et al., 2019), comprising Parkinson’s disease, dementia with Lewy bodies and multiple system atrophy. Both Parkinson’s disease and dementia with Lewy bodies are characterized by intercellular inclusion bodies, known as Lewy bodies (Shults 2006). The demonstration that the main component of Lewy bodies is a β-sheet-rich, fibrillar form of αS (Shults 2006; Araki, Yagi et al., 2019; Lashuel 2020), has motivated extensive studies of αS fibrils (Waxman and Giasson 2009; Alam, Bousset et al., 2019; Guerrero-Ferreira, Kovacik et al., 2020). It has also been shown that Lewy bodies contain membrane lipids (Lashuel 2020; Mahul-Mellier, Burtscher et al., 2020), which has motivated detailed studies on interaction between αS and lipids (Pfefferkorn, Jiang et al., 2012; Andreasen, Lorenzen et al., 2015; Iyer and Claessens 2019; Lashuel 2020), covering systems where the protein is present in the monomeric state (Jain, Bhasne et al., 2013; Fusco, De Simone et al., 2014; Fusco, Pape et al., 2016; Hannestad, Rocha et al., 2020), during the aggregation (Jiang, de Messieres et al., 2013; Galvagnion, Brown et al., 2016; Gaspar, Pallbo et al., 2018) as well as in the final amyloid aggregates (Hellstrand et al., 2013b; Galvagnion, Topgaard et al., 2019; Gaspar, Idini et al., 2021).

Amyloid fibrils often have very large aspect ratios, L/D > 100 (length over cross-section diameter). Considering that fibrils are sufficiently charged to be colloidally stable, the large aspect ratios allow fibrils to form an overlapping network in solution above a critical volume fraction (overlap concentration) $\phi^{*}\approx 10{\left(L/D\right)}^{-2}$ . For a typical protein, with a mass density of 1.4 g/cm^3^, L/D>100 means that the fibrils may form a network already for concentrations below 1.4 mg/ml. Thus, colloidally stable amyloid systems can form hydrogels already at very low protein concentrations (Frohm, Denizio et al., 2015; Pogostin, Linse et al., 2019).

In the case of αS, it has been shown that besides a long-range electrostatic repulsion, fibril-fibril interactions are also characterized by a short-range attractive interaction, presumably due to hydrophobic patches on the fibril surface (Semerdzhiev, Lindhoud et al., 2018; Pogostin, Linse et al., 2019). As the protein charge depends on the solution pH, the effective fibril-fibril interaction is hence pH dependent, and shifts from dominatingly repulsive to dominatingly attractive in the vicinity of the isoelectric point (pI ≈ 4.8 (Croke, Patil et al., 2011)) (Pogostin, Linse et al., 2019). Pogostin et al. (Pogostin, Linse et al., 2019) investigated αS fibril structure and interactions in pH range 5.5–7.5 and they found that the fibril structure, including its radius of 5.2 nm, was independent of the pH while the fibril-fibril interactions gradually switched from repulsive to attractive with decreasing pH. At pH = 5.5, the system no longer shows the property of a gel, indicating that the fibril network collapses into clusters.

The reason why these inclusions form *in vivo* are still not understood. We note, however, that such accumulations of aS fibrils, together with some other components, including lipids and other protein, are typically consequences of attractive interactions, suggesting that it could be of particular interest to study the behavior of αS fibrils under conditions when they are not colloidally stable. We achieved attractive, colloidally unstable αS fibrils under mildly acidic pH, close to the αS pI. In this study, we present a small angle neutron scattering (SANS) study of attractive αS fibrils formed at pH = 5.5, in pure buffer but also in the presence of different model lipid membrane systems. Small angle scattering is an ideal tool to study the arrangement of colloids on the 1–100 nm length scale (Glatter 2018), which includes amyloid fibrils (Ricci, Spinozzi et al., 2016), as it is non-destructive and experiments can be performed directly in a solution state.

## 2 Materials and Methods

### 2.1 α-Synuclein

Human αS was expressed in *E. coli* and purified as previously described in (Grey, Linse et al., 2011). αS monomers were isolated by size exclusion chromatography in 10 mM MES [2-(N-morpholino)ethanesulphonate] buffer at pH 5.5 using a 24 ml Superdex75 column (GE healthcare). Protein samples corresponding to the central region of the peak were then collected. The peptide concentration was determined by absorbance at 280 nm using an extinction coefficient 5,800 $\;M^{-1}cm^{-1}$ . To obtain high concentration required for scattering experiments, samples were lyophilized after size exclusion column.

*E. coli* cell pellet containing matchout deuterated αS was prepared in the Deuteration Laboratory of the Institut Laue-Langevin (ILL) in Grenoble, France as described by (Hellstrand et al., 2013a). A high cell density fed-batch culture using 85% deuterated Enfors minimal medium was carried out with computer-controlled temperature at 30°C and pO_2_ at 30% saturation (Haertlein, Moulin et al., 2016). The degree of deuteration was 75%. Deuterated αS monomers were isolated as described above.

### 2.2 Vesicle Preparation

The lipids used in this study were the phospholipids 1,2-dioleoylysn-glycero-3-phosphocholine (DOPC), 1,2-dioleoyl-sn-glycero-3-phospho-l-serine (DOPS), 1-palmitoyl-2-oleoyl-glycero-3-phosphocholine and (POPC), 1-palimtoyl-2-oleoyl-sn-glycero-3-phosho-l-serine (POPS), 1,2-dimyristoyl-sn-glycero-3-phosphocholine (DMPC), 1,2-dimyristoyl-sn-glycero-3-phospho-l-serine (DMPS), and the ganglioside lipids GM1 and GM3 from ovine brain. All lipids were obtained from Avanti Polar Lipids (Alabaster, AL, Unites States). In the preparation of mixed lipid vesicles, lipids were weighted and mixed with the desired proportion (PC:other 9:1). The powder was dissolved in chloroform:methanol (3:1 volume ratio) mixture. The solvent was evaporated under a stream of $N_{2}$ gas, and the lipid film was then dried in a vacuum oven over night. The lipids were finally dispersed in the desired buffer (10 mM MES buffer at pH 5.5) and vortexed for a few minutes.

Vesicles were formed either *via* sonication or extrusion. The sonication was performed for 15 min, 10 s on/off duty at 75% amplitude on ice. The lipid dispersions were centrifuged for 10 min at 1361 rad/s in order to pellet any contaminating particles from the sonicator tip. The supernatant was collected and used as the vesicle dispersion. Extruded vesicles were prepared using a 100 nm pore size filters with 21 passes in total.

### 2.3 Samples

In the present study, we analyze and discuss scattering data from fibrils formed at different conditions, in the presence of model membranes with various lipid compositions, obtained at different neutron scattering facilities. For simplicity, samples are numerically labelled and are described in the Table 1, grouped together according to the scattering facility at which the samples were measured, as the sample preparation was different for each facility. A more detailed description of the sample preparation and the SANS experimental conditions is provided in the following text. The buffer used for all samples was a 10 mM MES buffer at pH = 5.5.

**TABLE 1:** Summary of samples investigated. The table shows the protein and lipid concentration, lipid composition in model membranes and deuteration level of the buffer used in the scattering experiment (M = mol/L).

Sample number	Protein type and concentration	Buffer composition	Lipid composition	Lipid to protein molar ratio
1	d-αS, 110 μM	100% H_2_O	—	—
2	d-αS, 110 μM	100% H_2_O	DOPC/DOPS	1
3	d-αS, 110 μM	100% H_2_O	DOPC/GM1	1
4	d-αS, 110 μM	100% H_2_O	DOPC/GM3	1
5	h-αS, 140 μM	100% D_2_O	—	—
6	h-αS, 140 μM	100% D_2_O	DOPC/DOPS	0.4
7	h-αS, 140 μM	100% D_2_O	DOPC/GM1	0.4
8	h-αS, 140 μM	100% D_2_O	—	—
9	h-αS, 140 μM	100% D_2_O	DMPC/DMPS	1
10	h-αS, 140 μM	100% D_2_O	DMPC/DMPS	5
11	h-αS, 140 μM	100% D_2_O	DMPC/DMPS	15
12	h-αS, 140 μM	100% D_2_O	POPC/POPS	1
13	h-αS, 140 μM	100% D_2_O	POPC/POPS	2
14	h-αS, 140 μM	100% D_2_O	POPC/POPS	5
15	h-αS, 140 μM	100% D_2_O	POPC/GM1	1
16	h-αS, 140 μM	100% D_2_O	POPC/GM1	2
17	h-αS, 140 μM	100% D_2_O	POPC/GM1	5
18	h-αS, 140 μM	100% D_2_O	POPC/GM3	1
19	h-αS, 140 μM	100% D_2_O	POPC/GM3	2

#### 2.3.1 Samples 1–4

Samples 1–4 were composed of deuterated αS, alone or in the presence of protonated lipids, in 100% H_2_O buffer. The monomeric αS protein was incubated alone (sample 1) or mixed with a dispersion of sonicated vesicles: DOPC:DOPS (sample 2), DOPC:GM1 (sample 3), or DOPC:GM3 (sample 4). The protein and lipid concentrations were both 110 μ M with the lipid-to-protein molar ratio of 1.0. Samples were incubated in low-protein-binding tubes (Axygen) for 72 h at 37°C under stirring condition at 200 rpm.

After 72 h incubation the samples were centrifuged at 6,720 rcf for 2 min. The supernatant was separated from the sedimented fibrils and discarded. The separation of supernatant from the sediment was done to minimize the impact on the scattering profile of lipid residues that were not part of the aggregates and hence did not sediment during the centrifugation. Fibrils were then freeze-dried before transportation to the experimental site, where they were re-hydrated with buffer.

#### 2.3.2 Samples 5–7

Samples 5–7 were composed of protonated αS, alone or in the presence of protonated lipids, in 100% D_2_O buffer. The monomeric αS protein was incubated alone (sample 5) or mixed with a dispersion of sonicated vesicles: DOPC:DOPS (sample 6), or DOPC:GM1 (sample 7) in H_2_O buffer. The protein concentration was 140 μ M and lipid-to-protein molar ratio in samples six and seven were 0.4. The samples were incubated with stirring at 200 rpm in a low-protein-binding tubes (Axygen) for 72 h at 37°C. Samples were then dialyzed with 100% D_2_O buffer overnight with the aid of a dialysis membrane having $M_{w}$ cut off 3,500 kDa.

#### 2.3.3 Samples 8–19

Samples 8–19 were composed of protonated αS, alone or mixed with protonated lipids, in 100% D_2_O buffer. The monomeric αS protein was incubated alone (sample 8) or mixed with a dispersion of extruded vesicles DMPC:DMPS (samples 9–11), POPC:DOPS (samples 12–14), POPC:GM1 (samples 15–17), or POPC:GM3 (samples 18 and 19). The protein concentration was 140 μ M and different lipid-to-protein molar ratios in the range 0–15 were used. See Table 1 for details. Samples were incubated for 7 days at 37°C under quiescent conditions (samples 8–11), or for 5 days under stirring at 200 rpm (samples 12–19), in low-protein-binding tubes (Axygen).

After the incubation period, a 4-step washing procedure was performed on samples 9–19 prior to the SANS experiments. This was done in order to wash away lipids from the sample allowing to record only fibril scattering. The first step of the procedure is centrifugation at 15,615 rcf for 15 min, which resulted in a formation of a dense pellet. The supernatant above the formed pellet was removed in the second step of the procedure. Afterwards, the pellet was resuspended in the same amount of buffer as had been removed in the second step. The fourth step of the procedure involves redispersing the pellet by shaking and gentle vortexing. This procedure was repeated five times.

### 2.4 Small Angle Neutron Scattering Experiments

Small angle neutron scattering (SANS) experiments were carried out at three different facilities. Below we describe the experimental procedures for each set of experiments.

Samples 1–4 were measured at the D22 beam line located at Institut Laue-Langevin (ILL) in Grenoble France. Three different sample-to-detector distances, 17.6, 5.6, and 1.4 m, with collimation lengths of 17.6, 5.6, and 2.8 m, respectively, were combined. The neutron wavelength was 6.0 Å with the wavelength spread of 10%. Detector patterns were reduced using Grasp software (C. Dewhurst), including thickness and background, as well as direct flux normalization to obtain scattered intensity in absolute units. Scattering curves obtained at the different sample-to-detector distances were combined giving a total *q*-range comprised between 0.002 and 0.6 Å^−1^, where *q* is the wave vector transfer. Samples were measured in single stopper cylindrical cells 120-QS Hellma quartz cuvettes with a 1 mm path length. Measurements were taken at 37°C with the use of a rotating rack to prevent the sedimentation of the fibrils.

Samples 5–7 were measured at the LOQ beamline located at the ISIS Neutron and Muon Source, Chilton, United Kingdom. Samples were measured in single stopper cylindrical cells 120-QS Hellma quartz cuvettes with a 1 and 2 mm path length. A fixed sample-to-detector distance (4 m) combined with a white beam and time-of-flight detection provided a q range of 0.009–0.25 Å^−1^. The raw scattering data collected at the LOQ instrument in ISIS were corrected for the efficiency and spatial linearity of the detectors, the sample transmission and the background scattering using the instrument dedicated software Mantid (https://www.mantidproject.org/) and the standard procedure indicated in the software guide. Data were then converted into scattered intensity data I(Q). These data were then placed on an absolute scale (cm^−1^) by comparison with the scattering profile collected from a calibration standard, constituted of a solid blend of hydrogenous and perdeuterated polystyrene which has been measured with the same instrument configuration as per established procedures (Wignall and Bates 1987). Measurements were performed at 37°C with the aid of a rotating rack in order to prevent the sedimentation of the fibrils.

Samples 8–19 were measured at NG7 SANS instrument located at NIST Center for Neutron Research, Gaithersburg, MD, United States. Measurements were performed at four sample to-detector distances (1, 4, 13, and 15.3 m with lenses), and a neutron wavelength of 6.0 Å (sample-to-detector distances of 1, 4, and 13 m) and 8.1 Å (15.3 m with lenses), to obtain a *q* range spanning from 0.001 to 0.5 Å^−1^. The wavelength spread is approximately 12% (Glinka, Barker et al., 1998). The data was reduced to the absolute scale using the Igor software by following the standard protocol at NCNR to correct the effect of the background, empty cell, detector efficiency, and the transmission of each sample (Kline 2006). Samples 8–11 were measured in 2 mm path length demountable Ti cells with quartz windows, and samples 12–19 were measured in 1 mm path length banjo quartz cells. Measurements were performed at room temperature. The cells were mounted on a slowly rotating stage to prevent sedimentation during the experiment.

## 3 Results and Discussion

### 3.1 SANS Studies of αS Fibrils

The current work explores, with the use of SANS, the structural organization of αS fibrils at pH 5.5, which is close to their isoelectric point. In these conditions, the fibrils are not colloidally stable, but precipitate out of solution by aggregating into clusters that are prone to sediment (Pogostin, Linse et al., 2019). A total of 19 different samples, for simplicity labeled from 1 to 19, were investigated.

**FIGURE 1:**
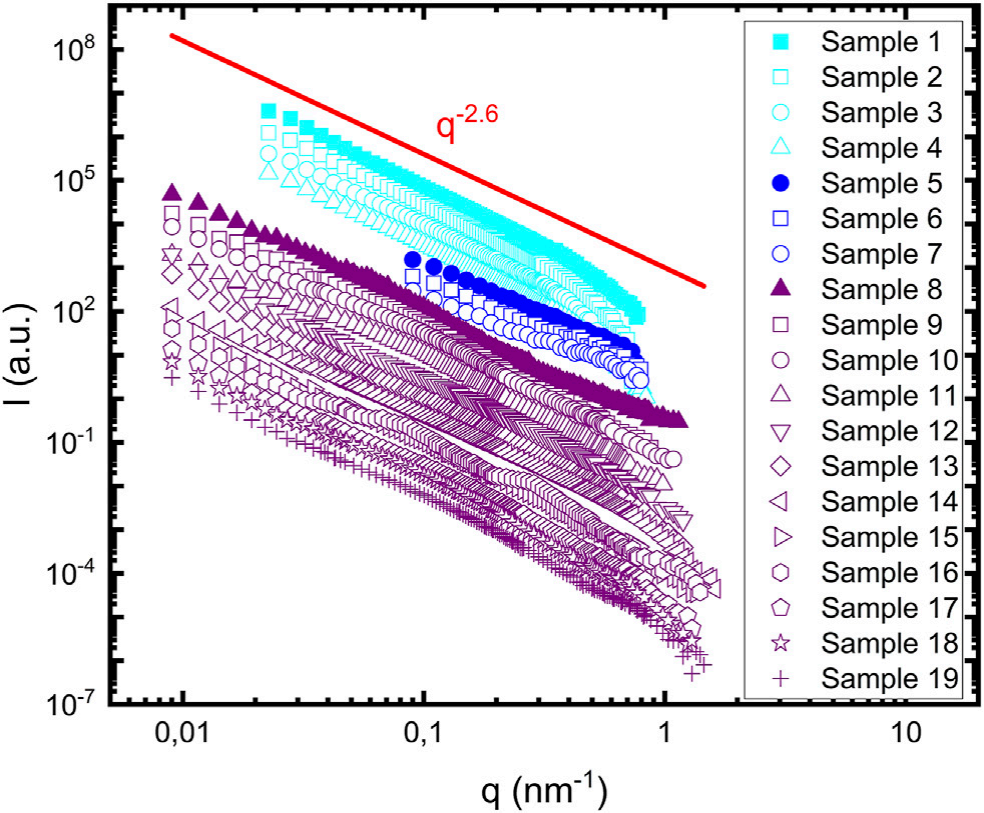
Scattering profiles of 19 samples summarized in Table 1. Samples containing lipids are represented with open symbols. Samples 1–4 are shown in **cyan**, samples five to seven are shown in **blue** and samples 8–19 are shown in **purple**. Samples containing protein alone are represented with filled symbols. The red line represents the power law dependence of the scattering profile, with a power value equal to 2.6.

Figure 1 shows SANS patterns, I(q), acquired from all 19 samples probed. The data are shifted with an arbitrary scale for better representation. The data on the absolute scale are shown in Supplementary Figure S1. As can be observed from Figure 1, the scattering patterns from the different samples are strikingly similar. They show a power law scattering, $I\left(q\right)∼q^{-d}$ , over essentially the full q -range covered by the experiments. All 19 data sets were fitted with a simple power law at lower q-values, giving a mean value 〈d〉=2.6 with standard deviation σ=0.3 (Supplementary Figure S2). A solid line illustrating d=2.6 is shown for comparison in Figure 1. We note that slight deviations from a perfect straight line can be observed for some of the samples, in particular sample number 16. One reason for the deviations could be that the clusters are not homogeneous fractal objects, but there are some heterogeneities and a slight variation of the fractal dimension with the probed length scale. At higher q-values there may also be a coupling with the fibril cross section form factor. However, these deviations are minor, in particular if we focus on low q regime, and the overall picture supports that we have fractal aggregates with an average fractal dimension of 2.6.

αS fibrils have the shape of homogeneous cylinders, with a radius R=5 nm (Pogostin, Linse et al., 2019). Long cylinders typically scatter as $I\left(q\right)∼q^{-1}$ (Pedersen 1997), at lower q -values (qR≪1) . The much steeper q -dependence observed here, $I\left(q\right)∼q^{-2.6}$ , is a signature of dominating attractive fibril-fibril interactions and that the fibrils aggregate further into fractal clusters, where the value d=2.6 can be interpreted as a fractal dimension (Lazzari, Nicoud et al., 2016). The value 2.6 is similar, but slightly larger than what is typically found for rod clusters (d=2.0−2.2) (Mohraz, Moler et al., 2004; Solomon and Spicer 2010). However, the exact value that reflects the fibril packing in the clusters, is expected to depend on the cluster formation mechanism (Murphy, Hatch et al., 2020), for example through diffusion-limited or reaction-limited cluster aggregation (Weitz et al., 1985; Lazzari, Nicoud et al., 2016). As a comparison, we note that one particular case of rigid-rod clusters corresponds to the case where randomly oriented rods are connected end-by-end, forming a chain. This case corresponds to the freely-jointed-chain (FJC) model used to describe semi-flexible polymers (Rubinstein and Colby 2003). It is also associated with the random-walk model of translational diffusion (Evans and Wennerström 1999) and is characterized by d=2.0 . The value d=2.6 observed here implies a denser packing compared to the FJC model.

### 3.2 Modeling of Fibril Clusters and Their Scattering

In order to better understand the fibril cluster organization, we have constructed fibril clusters using a simple fibril model. The approach by which individual fibrils are connected, is inspired by the FJC model. From the constructed fibril clusters, we calculate the corresponding scattering function, i.e., the cluster formfactor. Below, we present the model in detail and the way for calculating the scattering. As this is a new approach for describing rod clusters, that also may be used to analyze other rigid rod assemblies, we also analyze the model itself in some detail. To assess the present approach we analyze the model scattering function by comparing it with analytical Beaucage model (Hammouda 2010) of fractal objects.

**FIGURE 2:**
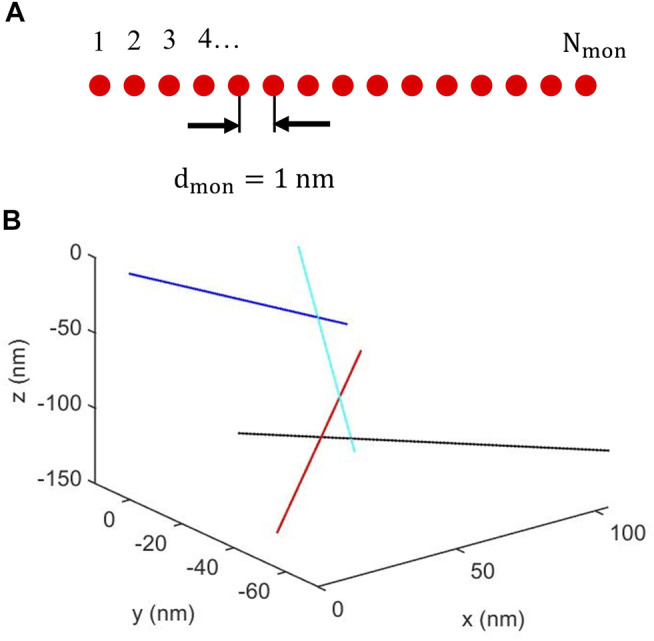
**(A)** Schematic illustration of a fibril composed of a linear array of N_mon_ identical monomers equally spaced with a separation d_mon_. **(B)** Example of cluster of four fibrils. For clarity, the fibrils are represented with different colors. Fibril #1 **(blue)** shares a monomer position with fibril #2 **(cyan)**. Fibril #2 also shares a monomer position with fibril #3 **(black)**, that in addition shares a monomer position with fibril #4 **(red)**.

In the present cluster model, individual fibrils were modeled as infinitesimally thin rods, represented by a straight line, of total *N*_*mon*_ point scatterers, referred to as monomers, that are separated by a distance *d*_*mon*_, as schematically illustrated in Figure 2A. Thus, the fibril length, *L*, is given by $L=\left(N_{\mathrm{mon}}-1\right)d_{\mathrm{mon}}$ . Fibril clusters were constructed by adding fibrils stepwise, with fibrils labeled from #1 to #*N*_*fib*_, where *N*_*fib*_ is the total number of fibrils in the cluster. First, fibril #1, having a randomly chosen orientation, was constructed. Then, fibril #2, again with a randomly chosen orientation, was constructed. A randomly chosen monomer of fibril #2 was given the same position [(x,y,z) coordinate] as one of the monomers of fibril #1, that was also randomly chosen. The process of adding fibrils having random orientation continued, with fibril #3 connecting to fibril #2 and fibril #4 connecting to fibril #3 etc. Finally, a cluster was completed with fibril #*N*_*fib*_ connecting to fibril #(*N*_*fib*_ − 1). As an illustration, a system with *N*_*fib*_ = 4 and *N*_*mon*_ = 100 is depicted in Figure 2B.

The spherically averaged scattering intensity, *P*_*c*_(*q*), from the cluster, was then calculated from the spherically averaged Debye scattering equation (Farrow and Billinge 2009)

$$P_{c}\left(q\right)=\sum_{i=1}^{N}\sum_{j=1}^{N}\frac{\sin\left(qr_{ij}\right)}{qr_{ij}}.$$ (1)

Here, $r_{\mathrm{ij}}=\left|\vec{r_{i}}-\vec{r_{j}}\right|$ with $\vec{r_{i}}$ and $\vec{r_{j}}$ being the positions of monomers *i* and *j*, respectively. The double sum runs over the total number, *N*, of monomers in the cluster, $N=N_{\mathrm{mon}}N_{\mathrm{fib}}$ , treating all monomers as identical point scatterers. Eq. 1 represents a single cluster scattering function, i.e., the cluster form factor *P*_*c*_(*q*). A cluster, generated by the process described above, is unique and represented by a unique function *P*_*c*_(*q*). Thus, in order to form a proper ensemble average, a sum over a number, *N*_*c*_, of clusters were performed to obtain the ensemble averaged $\langle P_{c}\left(q\right)\rangle$ .

**FIGURE 3:**
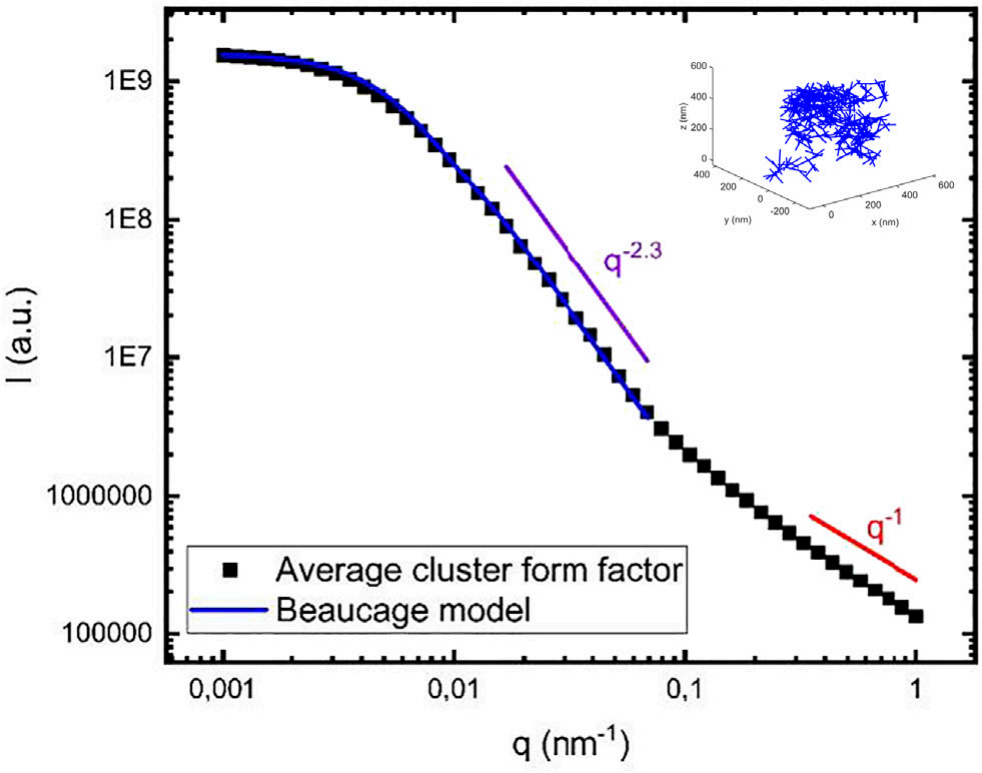
Calculated cluster form factor obtained from averaging over 20 different clusters, each containing 400 fibrils **(black squares)**. As an example, one of the modeled clusters is shown in the inset. The **blue** line is a calculated scattering curve using the Beaucage model **(see text)** with $G=1.6\;10^{-9}$ , Rg=300 nm and d=2.3 . As a **red** line we show $q^{-1}$ dependence of the scattering intensity expected at high q and representing the single rod form factor, and as a **purple** line we show $q^{-2.3}$ dependence of the scattering intensity.

Figure 3 displays the scattering pattern $\langle P_{c}\left(q\right)\rangle$ obtained by averaging data from *N*_*c*_ = 20 simulated clusters, each with *N*_*fib*_ = 400, *N*_*mon*_ = 100 and *d*_*mon*_ = 1 nm. *N*_*fib*_ and *N*_*mon*_ were chosen to have reasonable computing times (≈1.5 h per cluster) on a normal PC. To confirm that *N*_*c*_ = 20 was sufficient to obtain a reasonable ensemble average, we compare with different averages taken with lower values of *N*_*c*_ in Supplementary Figure S3. We conclude that averaging over 10 clusters already results in reproducible scattering profiles.

The model cluster involve two characteristic length scales, the overall cluster radius of gyration, $R_{g}$ , and the mesh size, ξ , within the fibril network. Thus, the scattering pattern in Figure 3 can be divided up into three regimes (Solomon and Spicer 2010). At lower q -values, $q<1/R_{g}$ , there is the Guinier regime, where the scattered intensity is given by $I\left(q\right)=I\left(0\right)\exp\left(-\frac{q^{2}R_{g}^{2}}{3}\right)$ . In the intermediate *q*-range, $1/R_{g}<q<1/\xi$ , the scattered intensity takes a power law $I\left(q\right)∼q^{-d}$ , where d corresponds to the cluster fractal dimension. Finally, for q>1/ξ we have $I\left(q\right)∼q^{-1}$ , which is the high q form factor of the (infinitely thin) model fibrils. The full single fibril form factor, for *N*_*mon*_ = 100 and *d*_*mon*_ = 1 nm, is shown in the Supplementary Figure S4.

In Figure 3, the simulated scattering curve is also compared with the analytical Beaucage model (Hammouda 2010). Beaucage model describes fractal objects, and it has been used to describe amyloid fractals formed by amyloid-β, a protein involved in Alzheimer’s disease (Festa et al., 2019a; Festa et al., 2019b). The model describes a low *q* Guinier regime, followed by a Porod regime with a power law q -dependence of the intensity, *q* ^*-d*^, for $q>1/R_{g}$ , $R_{g}$ again being the radius of gyration. Thus, this model has three independent parameters, $R_{g}$ , the fractal dimension, *d*, and a scale factor for the intensity. The model scattered intensity is given by

$$I_{B}\left(q\right)=G\exp\left\{-\frac{q^{2}R_{g}^{2}}{3}\right\}+\frac{C}{q^{d}}{\left(\mathrm{erf}\left\{\frac{qR_{g}}{\sqrt{6}}\right\}\right)}^{3d},$$ (2)

Here, erf(x) is the error function and the so called Porod scale factor, C , is related to the Guinier scale factor G by

$$C=\frac{Gd}{R_{g}^{d}}{\left(\frac{6d^{2}}{\left(2+d\right)\left(2+2d\right)}\right)}^{d/2}\Gamma \left(\frac{d}{2}\right),$$ (3)

where Γ(x) is the gamma function. In the calculated curve shown in Figure 3, we have used $G=1.6\;10^{9}$ [here, $G={\left(N_{\mathrm{fib}}N_{\mathrm{mon}}\right)}^{2}$ ], $R_{g}=300\;\mathrm{nm}$ and d = 2.3 . From the crossover to $q^{-1}$ in Figure 3, we estimate ξ≈10 nm .

Experimentally we observe d=2.6 indicating slightly more dense clusters than what is produced by the simple model above. In the present model, the cluster is essentially a chain of fibrils, where each fibril is connected to two other fibrils. This construction is related the freely jointed chain (FJC) model of polymers (Rubinstein and Colby 2003), which in turn is associated with the random walk model of translational diffusion. For our conceptual understanding of what influences the fractal dimension, it is interesting to compare quantitatively with the FCJ model. In our cluster model the fibrils connect at randomly chosen monomer positions. In the FJC model, on the other hand, *N* rigid-rod segments, of length *l* (the Kuhn length), are connected end-to-end. The radius of gyration of such a chain is given by (Rubinstein and Colby 2003)

$$R_{g}={\left(\frac{1}{6}Nl^{2}\right)}^{1/2},$$ (4)

and the fractal dimension d = 2 (Rubinstein and Colby 2003). Equation 4 holds only strictly for the FJC model. However, we can still use it to estimate *R*_*g*_ for our model cluster. In our model, we need to consider an average (random walk) step length, <*l*>, that here can be identified with the average separation between the two monomer positions within a fibril that are shared with other fibrils. Thus, *l* is limited to 1 ≤ *l*/*d*_*mon*_ ≤ (*N*_*mon*_ − 1). The probability of a given *l*-value deceases monotonically with increasing *l*. Defining *l*/*d*_*mon*_ = *n*, the number of monomers between two connections, we have

$$\langle l\rangle /d_{\mathrm{mon}}=\frac{\sum_{n=1}^{N_{\mathrm{mon}}-1}n\left(N_{\mathrm{mon}}-1-n\right)}{\sum_{n=1}^{N_{\mathrm{mon}}-1}\left(N_{\mathrm{mon}}-1-n\right)},$$ (5)

With *N*_*mon*_ = 100 and *d*_*mon*_ = 1 nm, we obtain <*l*> = 33 nm. With this value of <*l*> and *N* = *N*_*fib*_ = 400 in Eq. 4 we obtain $R_{g}=270\;\mathrm{nm}$ which is only slightly smaller than the value 300 nm obtained for the model clusters described above (Figure 3).

**FIGURE 4:**
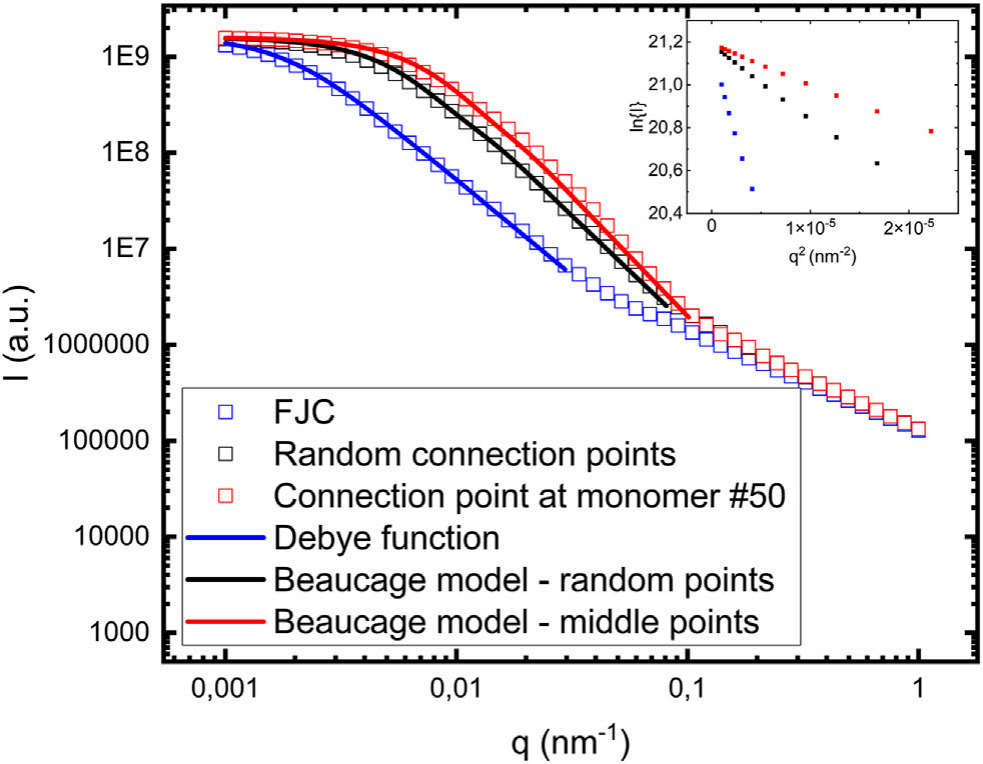
Comparison between different calculated cluster form factors: i) FJC model **(blue empty squares)**, ii) random connection points **(black empty squares)**, and iii) each fibril having one connection point at monomer #50 **(red empty squares)**. The **blue solid line** in i) correspond to a model calculation using the Debye function (Eq. 6). The **black and the red solid lines** correspond to model calculations using the Beaucage model (Eqs 2 and 3). *R*_*g*_ decreases and *d* increases from i) to iii). The inset shows Guinier plots ln(I) vs *q* ^2^ for the low *q* data, from which *R*_*g*_ also can be determined.

Within this simple cluster model, reducing the effective step length *l*, by connecting the fibril segments randomly reduces $R_{g}\;$ and increases *d*, compared to the limiting FJC case. In an attempt to decrease *l* further, and thereby possibly increase *d*, we also simulated clusters where we let fibril #(i+1) connect at the middle monomer (#50) of fibril #i, while the other parameters of the connections were randomly chosen. This decreases the maximum possible value of *l* from *N*_*mon*_ (100) to *N*_*mon*_/2 (50). The calculated scattering pattern from such clusters is shown in Figure 4. By again fitting the calculated scattering curve with the Beaucage model we obtain $R_{g}=220\;\mathrm{nm}$ and *d = 2.5*, using *G* = 1.6 10^9^

In Figure 4 we are also comparing with the FJC model, where fibrils are connected end-to-end. For ideal chains, like FJC chains, the form factor was derived by Debye (Debye 1947) and is consistent with *d* = 2

$$P_{\mathrm{FJC}}\left(q\right)=2\left(e^{-x}+x-1\right)/x^{2},$$ (6)

where *x* = *qR*_*g*_. Shown in Figure 4 as a solid blue line is a calculated model form factor P_FJC_(q) (Eq. 6) using Rg = 800 nm. This is in good agreement with Rg = 808 nm, calculated from Eq. 4.

As seen in Figure 4, *Rg* decreases and *d* increases as the distance between connection points in a fibril with neighboring fibrils is decreasing. Shown as an inset in Figure 4 are Guinier plots, ln(I(q)) vs q^2^ using data at low-q, from which we can do a model free evaluation of *Rg* from $\ln\left(I\left(q\right)\right)=-q^{2}R_{g}^{2}/3$ . The *Rg* values obtained this way are 690 nm for the case of FJC model, 300 nm for only random connection points and 240 nm for the case with one of the connection points being in the middle of the fibril. The values are in good agreement with the values obtained with the Debye and Beaucage model, respectively.

With the calculations presented above we demonstrate that within the simple model used, it is possible to construct fibril clusters having different fractal dimensions, including the values that we observe experimentally. Our experimentally observed value 2.6 is similar, but slightly larger than what is typically found for rod clusters ( d=2.0−2.2 ) (Mohraz, Moler et al., 2004; Solomon and Spicer 2010). However, the exact value, that reflects the fibril packing in the clusters, is expected to depend on how the clusters are formed for example through diffusion limited or reaction limited aggregation (Weitz et al., 1985; Lazzari, Nicoud et al., 2016).

In the experimental scattering patterns (Figure 1) we essentially observe only a single power law dependence of the scattered intensity $I\left(q\right)∼q^{-d}$ within studied *q*-range. Thus, we only observe one (the middle one) out of the three different q-regimes of the scattering pattern, discussed in connection with Figure 3. That we do not observe a Guinier regime with a leveling off of the scattered intensity at lower q -values implies that the formed clusters are much larger than $q_{\min}^{-1}\approx 100\;\mathrm{nm}$ , where *q*_min_ is the minimum q -values accessible in the experiment. Neither at higher q-values do we observe any crossover to $q^{-1}$ , related to a length scale where the one-dimensional nature of the individual fibril morphology would be detected. This implies a very dense packing of the fibrils in the clusters, with the mesh size being not much larger than the fibril diameter (10 nm).

The model that we have used in Figures 3, 4 assumes infinitely thin fibrils. The mesh size of such clusters is approximately equal to 10 nm. To further illustrate the effect of a finite size cylinder, we have extended calculation, and we are showing them in the Supplementary Figure S5.

### 3.3 αS Clusters in Biology

αS fibrils are a major component of Lewy bodies, a pathological feature of Parkinson’s disease (Shults 2006; Araki, Yagi et al., 2019; Lashuel 2020). They are micrometre sized intracellular inclusions in the *substantia nigra*, that also contain lipids, membranous organelles, as well as other proteins (Lashuel 2020; Mahul-Mellier, Burtscher et al., 2020). Agglomerates and clusters of this kind are typically consequences of dominating attractive interactions, and recent work has indicated that the accumulations of various species through an effective liquid-liquid phase separation may be effective in various biological functions (Hyman, Weber et al., 2014). Colloidal interactions in the living cell, e.g., protein-protein interactions and protein membrane interactions are typically weakly repulsive, because essentially all colloidal aggregates and macromolecules carry a net negative charge. This ensures the colloidal stability of the living cell (Wennerström, Vallina Estrada et al., 2020). An interesting question concerns the origin of, and the reason for, the effective attractive interaction resulting in the accumulation of αS fibrils, and other components, that lead to the formation of Lewy bodies. Here, in combination with a previous work (Pogostin, Linse et al., 2019), we have shown that a pH drop from neutral to mildly acidic conditions (pH = 5.5) is sufficient to switch fibril-fibril interactions from being predominantly repulsive to become predominantly attractive resulting in a dense clustering of αS fibrils. At the same time, the rate of αS fibril formation is significantly increased at mildly acidic pH (pH = 5.5) due to strongly enhanced secondary nucleation (Cohen, Linse et al., 2013).

Mildly acidic pH is indeed found in some cellular compartments such as lysosomes and also in endosomes (Demaurex 2002; Hu, Dammer et al., 2015). Attractive fibril-fibril interactions may also result from cleavage of the acidic C-terminus, which up-shifts the isoelectric point, or from increased salt screening of long-range electrostatic repulsion. The use of mildly acidic pH to induce fibril clustering likely mimics these three cases and may provide a route towards the study of fibril organization in Lewy bodies.

### 3.4 Summary and Conclusion

Dispersions of αS fibrils formed at pH 5.5 behave significantly different compared to those formed at slightly higher pH where a stable fibril hydrogel network can be formed. (Pogostin et al., 2019). At pH 5.5 the formed αS fibrils are colloidally unstable and aggregate further into clusters. Inspired by the fact that Lewy bodies appear to contain accumulations of αS fibrils, indicating effectively attractive fibril-fibril interactions, we have here investigated αS fibrils clusters at pH = 5.5 in more detail. SANS experiments performed on 19 different samples show strikingly similar result. The SANS intensities show an extended power law dependence on the scattering vector, *q*, that is consistent with that the clusters can be described as mass fractals, with a fractal dimension *d* ≈ 2.6. To further conform this conclusion, we have developed a simple model of rigid rod clusters, that was found to be able to reproduce the experimentally observed fractal dimension. The simple cluster model is closely related to the classical FJC model of polymers, that may also serve as a reference case with *d* = 2.

## Data Availability

The raw data supporting the conclusions of this article will be made available by the authors, without undue reservation.
