# Supplementary material for: On the Cluster Formation of α-Synuclein Fibrils
Source: Front Mol Biosci. 2021 Oct 19;8:768004. doi: 10.3389/fmolb.2021.768004 (PMC8560691; doi:10.3389/fmolb.2021.768004)
Supplement: Supplementary file 1 [file DataSheet1.docx]

Supplementary Material

# Absolute scale

# In the Figure S1 we are showing all 19 samples on the absolute scale, normalized by protein volume fraction. The difference in the intensity is observed in some of the samples, which can be explained by the difference in the amount of protein that fibrillated in different cases or by uncertainty caused by imperfect sample rotation during SANS measurements.


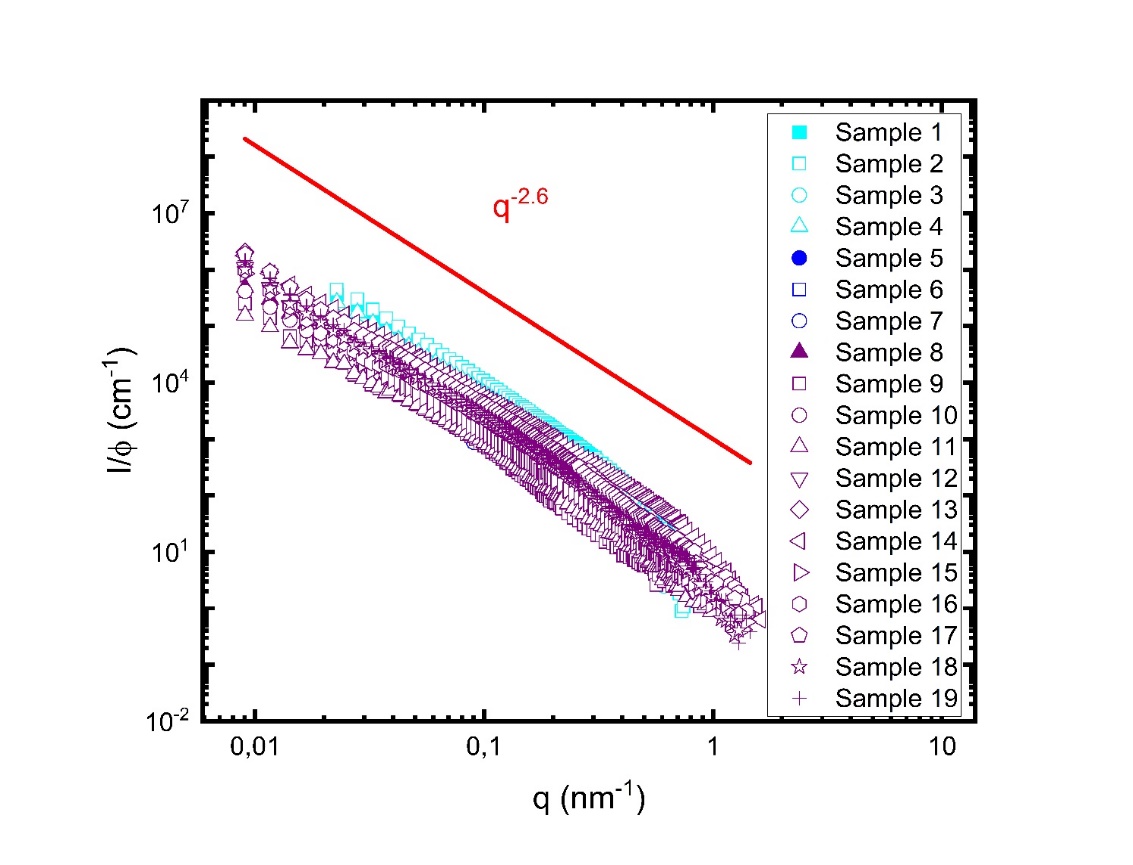


**Figure S1.** Scattering profiles of 19 samples presented on absolute scale, normalized by protein volume fraction. Samples containing lipids are represented with open symbols. Samples 1-4 are shown in cyan, samples 5-7 are shown in blue and samples 8-19 are shown in purple. Samples containing protein alone are represented with filled symbols. For comparison, as a red line is shown a power law dependence of the scattering intensity on q-vector, with power value equal to 2.6.

# The power-law exponent

The variation of the experimentally determined fractal dimension, d, in the 19 different samples, is presented in Figure S2 as a plot of the occurrence (number of observations) vs. d. The fractal dimension was obtained from a power-law fit to the scattering data at lower q-values.


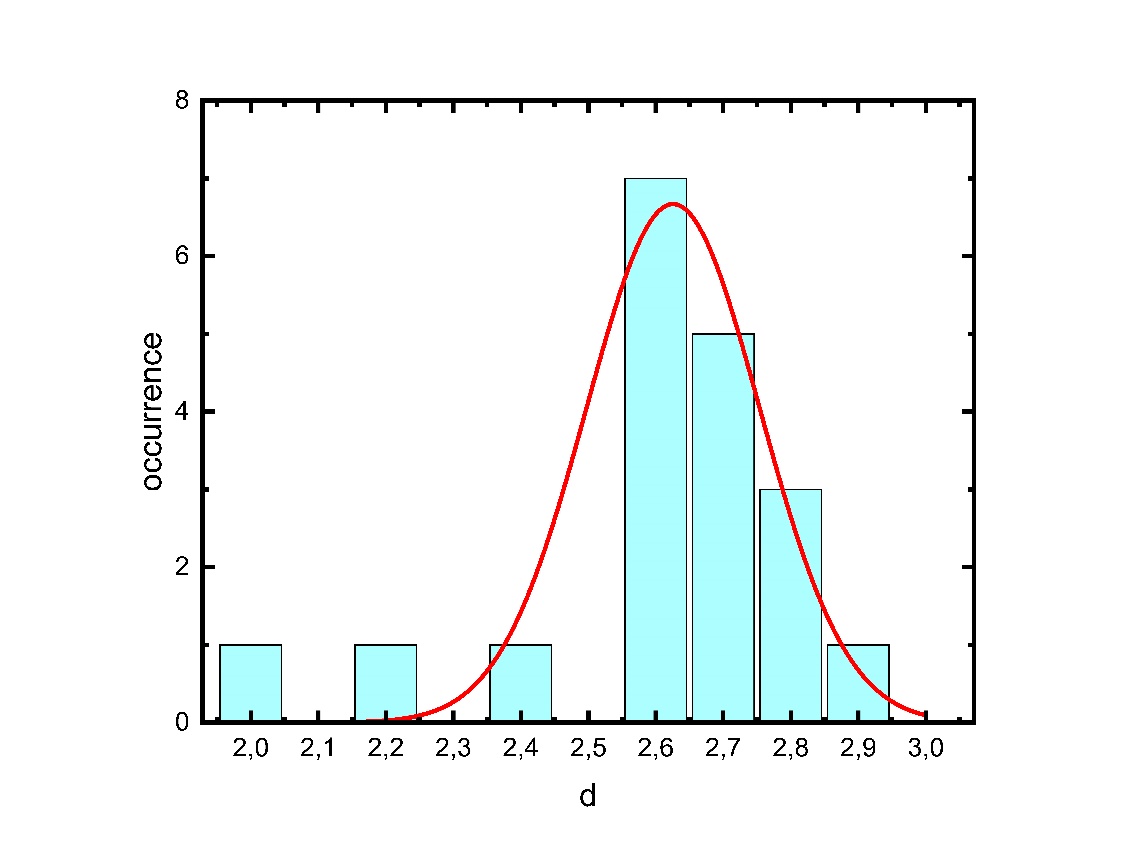


**Figure S2.** Bar chart illustrating how often does the value of power law between 2 and 3 occur in the data included in figure 2 of the main text and figures S2 and S3. The Gaussian fit (red line) gives standard deviation equal to 0.3 and the mean value equal to 2.6.

# Fibril cluster modeling

In Figure S3 we show calculated model scattering profiles from single clusters composed of 400 fibrils (panel a) and scattering profiles obtained by averaging over 5 (panel b) and 10 (panel c) such clusters. What is evident is that the individual clusters are unique, having their unique form factors. However, averaging over 10 clusters is sufficient to generate a reasonable ensemble average. The simulated scattering curve presented in Figure 3 was obtained by averaging over all 20 clusters.

In Figure S4 we show the simulated form factor of a single fibril (filled black squares). In this figure we also compare with the analytical form factor, *P_cyl_*(*q*) of an infinitely thin cylinder (solid blue line) given by ^1^

$$P_{cyl}\left( q \right)=\frac{2\mathrm{Si}\{qL\}s}{qL}-4\sin^{2} \{qL\}/{(qL)}^{2}$$

where $\mathrm{Si}\left\{ x \right\}=\int_{0}^{x} t^{-1}\sin\left\{ t \right\}dt$ and *L* is the cylinder length. In our case *L*=100 nm. As can be seen, the simulated formfactor is in good agreement with the analytical expression.


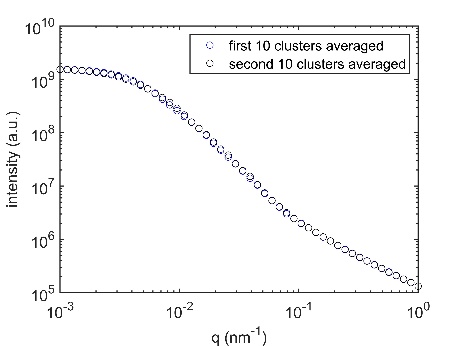

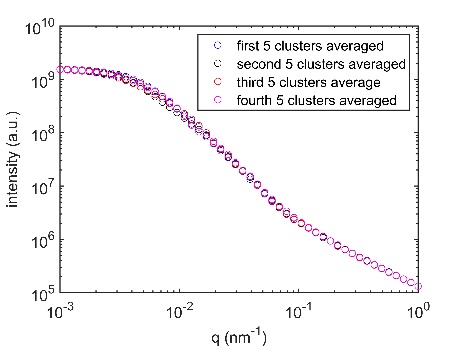

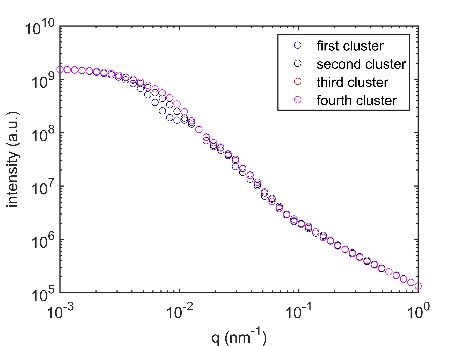


a) b) c)

**Figure S3.** Simulated scattering profiles of clusters composed of 400 fibrils containing 100 monomers separated by 1 nm distance. a) Comparison of single cluster scattering; b) Comparison of scattering profiles obtained by averaging over 5 clusters; c) Comparison of scattering profiles obtained by averaging over 10 clusters.


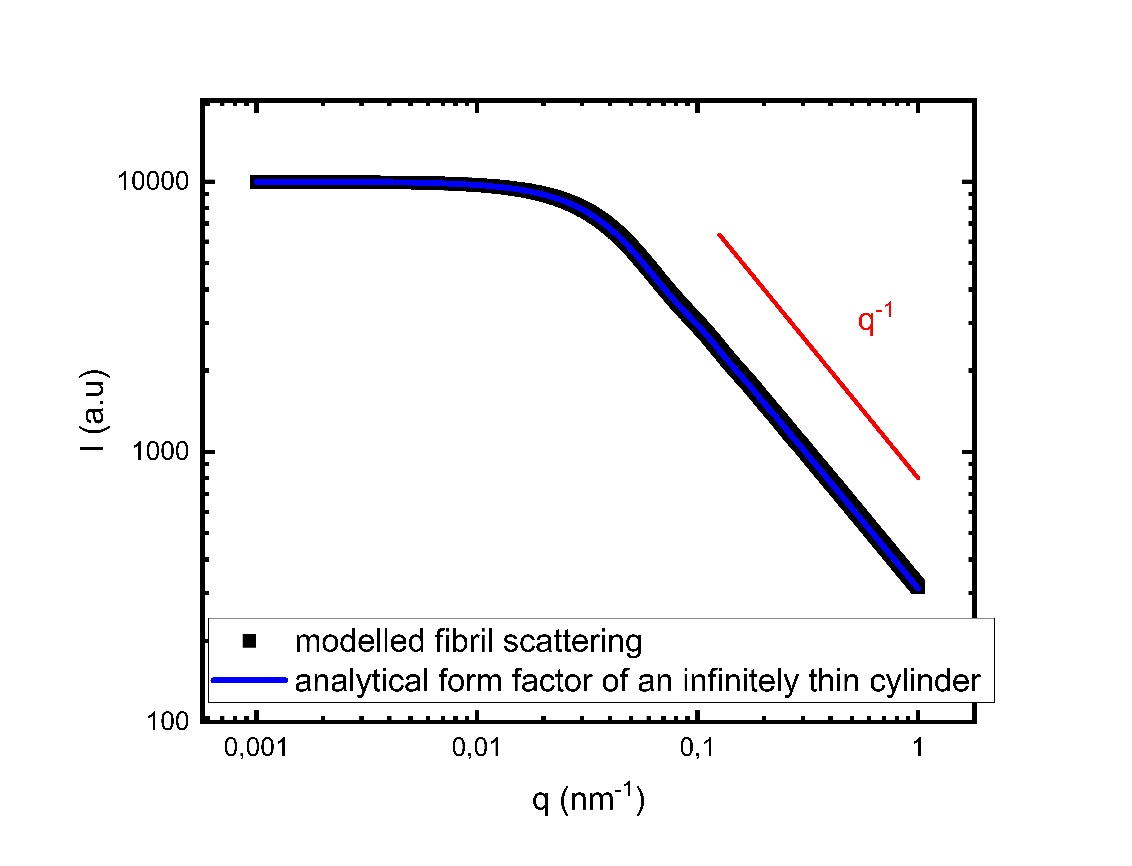


**Figure S4.** The simulated scattering of a single fibril (black squares), analytical form factor of an infinitely thin cylinder of 100 nm length (blue line) and the q^-1^ dependence of the scattering intensity (red line).

In the model discussed above, the fibril radius for simplicity is infinitely small, whereas the real fibrils have a diameter of ca. 10 nm ^2^. As concluded in the main manuscript, the fibril clusters appear very dense, as an extended regime with *I(q)~q^-1^* is not observed in the SANS pattern. Rather, we observe *I(q)~q^-d^*, with *d ≈ 2.6*, all the way to the *q*-regime where we expect to see a cross-over to a Porod regime, *I(q)~q^-4^*, associated with the fibril cross-section formfactor *P_cs_(q)*. In the model cluster presented in Figures 3 the mesh size is in fact $\xi$≈*10 nm*, similar to the real fibril diameter. Hence, one way to approximate a dense cluster with finite diameter fibrils is by simply multiply the model cluster formfactor *P_c_(q)* with a normalized cylinder cross-section form factor of a cylinder, given by^3^

$P_{cs}\left( q \right)=\left( 2\frac{J_{1}(qR)}{qR} \right)^{2}$ (S2)

where *R* is the cross-section radius. *P_cs_ (0)=1* and *P_cs_ (q)* remains ≈1 up to $q\approx1/R$ above which it decreases steeply with increasing *q*. The resulting product

$I\left( q \right)=P_{c}(q)P_{cs}(q)$ ( S3 )

is plotted in Figure S5.


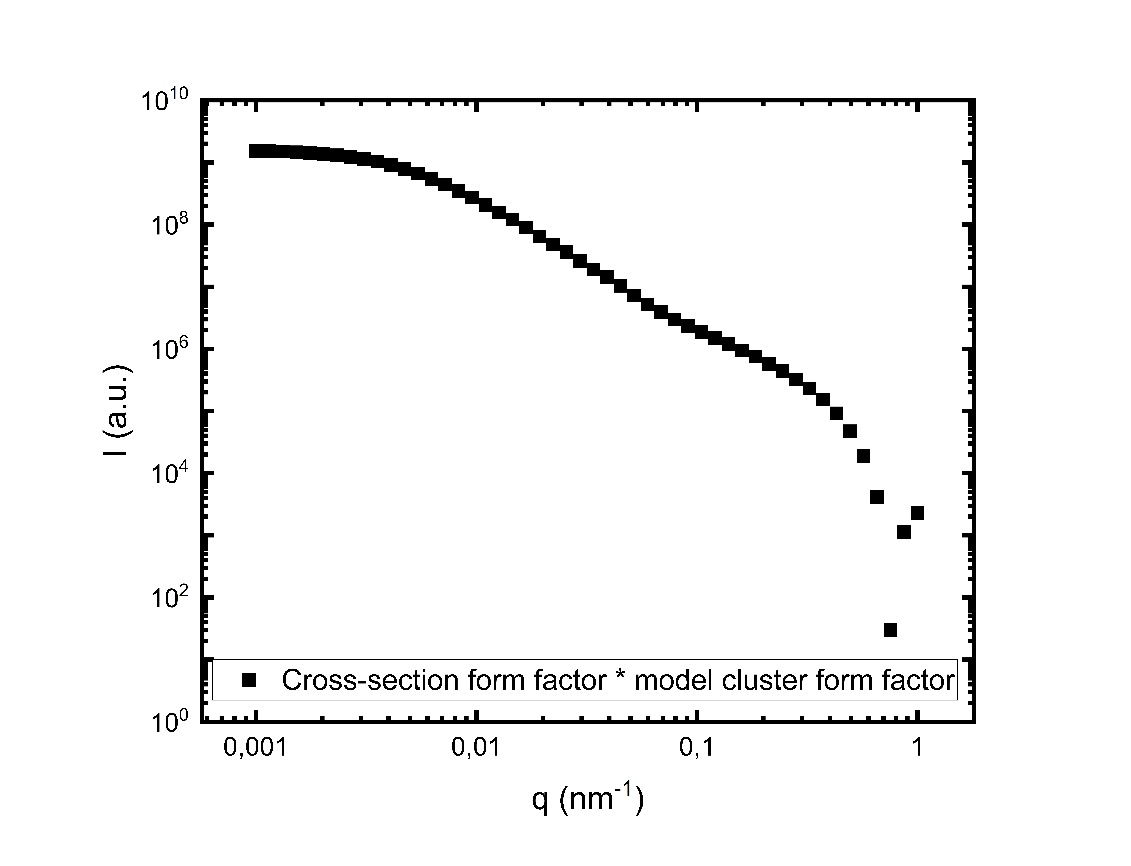


**Figure S5.** Modelled cluster form factor multiplied by normalized cross-section form factor of a cylinder (Eq. (S3)).

As can be seen in Figure S5, the region of *q^-1^* decay has now almost vanished, more resembling the experimental data at higher *q*-values. Although we are here essentially allowing for the interpenetration of the model fibrils, and the model should therefore be used with care, Eq. (S3) still allows for a simple approximation for treating a finite fiber cross-section.

1 Pedersen, J. S. Analysis of small-angle scattering data from colloids and polymer solutions: modeling and least-squares fitting. *Advances in Colloid and Interface Science* **70**, 171-210, doi:10.1016/s0001-8686(97)00312-6 (1997).

2 Pogostin, B. H., Linse, S. & Olsson, U. Fibril Charge Affects alpha-Synuclein Hydrogel Rheological Properties. *Langmuir* **35**, 16536-16544, doi:10.1021/acs.langmuir.9b02516 (2019).

3 Glatter, O. *Scattering Methods and Their Application in Colloid and Interface Science*. (Elsevier, 2018).
